# How To Advance Open International Scientific Exchange

**DOI:** 10.1371/journal.pcbi.1000097

**Published:** 2008-06-27

**Authors:** Barbara Bryant

**Affiliations:** Millennium Pharmaceuticals, Cambridge, Massachusetts, United States of America

Computational biology is an international collaboration. Open scholarly exchange nurtures the development of our field. And scientists are not the only beneficiaries; international cooperation is a crucial part of any country's diplomatic relations. Our community, by actively engaging governments, needs to promote scientific exchange.

In response to reports of visa difficulties, the International Society for Computational Biology (ISCB; http://www.iscb.org/) recently surveyed its members about the experience of non-US scientists visiting or working in the United States (http://www.iscb.org/US_visa_survey.html). The answers of 50 people of 20 nationalities revealed needless inefficiencies and problems. The results of the survey were shared with the National Academy of Sciences (NAS, http://www.nationalacademies.org/) in advance of a February 7, 2008, Senate hearing on the topic of barriers to scientific exchange (http://science.house.gov/publications/hearings_markups_details.aspx?NewsID=2064).

While ISCB collected information only on US visa problems, enabling international scientific exchange is a matter of importance in all countries. A 2005 European Union (EU) directive designed to make it easier for non-European scientists to get working visas for the EU has been implemented by only some member countries (http://www.euractiv.com/en/science/member-states-hesitant-welcome-foreign-researchers/article-167646, http://wbc-inco.net/news/3285.html). Visa agreements between countries are almost always reciprocal. For example, if China allows only single entry visas to US citizens, then the US allows only single entry visas to Chinese citizens. There is a need for all countries to work out better reciprocity agreements with each other in the name of international exchange and collaboration.

Concerns about terrorism negatively affected the US visa process; one result was that in the two years following the September 11, 2001, attacks, the number of foreign graduate students in the US fell significantly [1]–[3]. After 2003, this trend was reversed as policies were changed, but the situation is still not ideal, as reflected in the ISCB survey.

Too many of our fellow computational biologists have had to put their careers on hold, in some cases for more than 6 months, while waiting for permission to enter the US for study or a job. For some, permission arrives too late; the conference is over, or the research opportunity lost. For others, permission is never granted, with reasons either unknown or incomprehensible. Many non-US citizens living in the US are afraid to leave to visit family or attend a meeting, because getting back into the US can take a long time and is not guaranteed. Most survey respondents complained of the delays and lack of transparency in the visa process.

While many scientists have experienced positive interactions with helpful embassy and border personnel, there are also stories of ill treatment. Our colleagues have reported being spoken to rudely, detained at the border, interrogated, and even shackled. Some are afraid to tell their stories without assurance of anonymity, for fear of negative impact on their careers.

Perhaps most troubling of all are the many thousands of scientific collaborations and personal relationships that never had a chance to develop because scientists did not even attempt to come to the US as a result of these difficulties. The current situation is puzzling and disappointing given the central role that international scientists have played in enriching the US both culturally and economically.

Respondents to the ISCB survey gave recommendations, which we support, for the various stakeholders: government, scientific organizations, and scientists themselves.

Some of the recommendations for the US government are also applicable to other countries:

- Treat visiting scientists with respect and recognize their contributions.
- Have well-documented and transparent immigration policies.
- Make the immigration procedure predictable with fixed time lines.
- Streamline and simplify the visa process and reduce turnaround times.
- Make visas valid for a longer time; allow multiple entries.

Additional recommendations specific to the US government were to:

- Make it easier for non-US citizens living in the US to leave for a short time for meetings or visits home.
- Make it possible to extend the J-1, O-1, and other visas from within the US.
- Remove the 2-year 221(e) restriction.
- Perform the necessary MANTIS searches before the consular appointments.

Professional societies and conference organizers should:

- Educate embassy decisionmakers about our field, to reduce the mistaken tagging of computational biologists as security risks. Give scientists advice about how to explain what they do in such a way to make it clear that they are not a security risk.
- Notify submitters to conferences earlier of acceptances, to allow enough time to go through the visa process.
- Give full refunds to attendees not able to make it due to visa problems.
- Lobby government for changes listed above.
- Provide direct help to conference attendees in processing their visas, including explaining what documents are necessary.

What can you as a scientist do? A lot!

You can report US-visa–related issues to the NAS International Visitors Office (http://www7.nationalacademies.org/visas/Visa_Questionnaire.html). This information is used to support international scientific exchange, and in some cases to help address specific cases.

Talk and write to your legislative representatives as well as to all levels of government. Let them know about problems, and about the value of international scientific exchange to your work. Advocate specific improvements to policies, laws, and international agreements.

Work through scientific organizations such as ISCB, NAS, and FASEB (the Federation of American Societies for Experimental Biology, http://opa.faseb.org/). Many of these organizations have been very active in the issue of international scientific exchange.

And finally, share your experience, concerns, and ideas with other scientists. PLoS invites you to use the comments feature associated with this article to share your experience of traveling internationally, and your ideas about how to take action on this topic.

Enabling open scientific exchange is an important issue to the future of our science; your voice is important!
